# Draft Genome Sequences of Three Antibiotic-Producing Soil Bacteria, Staphylococcus pasteuri WAM01, Peribacillus butanolivorans WAM04, and Micrococcus yunnanensis WAM06, with Growth-Inhibiting Effects against Commensal *Neisseria* Strains

**DOI:** 10.1128/mra.00627-22

**Published:** 2022-09-12

**Authors:** Emily Reilly, Juan A. Alfaro, Alexis R. Borzelleri, Emma G. Branco, Declan C. Conklin, Emmaly S. Held, Fio Z. Kulee, Amelia J. Kuzma, Nic Langdon, Alyssa M. Lasko, Sean T. Neri, Jasmine A. Nichols, Temitope R. Olawuyi, Eunice Park, Kadrian Rugullies, Carter D. Wilkie, Laura R. Krebs, Dawn Carter, André O. Hudson, Crista B. Wadsworth

**Affiliations:** a Rochester Institute of Technology, Thomas H. Gosnell School of Life Sciences, Rochester, New York, USA; Wellesley College

## Abstract

We report the isolation, identification, and assemblies of three antibiotic-producing soil bacteria (Staphylococcus pasteuri, Peribacillus butanolivorans, and Micrococcus yunnanensis) that inhibit the growth of *Neisseria* commensals in coculture. With pathogenic *Neisseria* strains becoming increasingly resistant to antibiotics, bioprospecting for novel antimicrobials using commensal relatives may facilitate discovery of clinically useful drugs.

## ANNOUNCEMENT

Antibiotic resistance (AR) in Neisseria gonorrhoeae, the Gram-negative pathogen responsible for the sexually transmitted infection gonorrhea, is a worldwide threat to public health. Resistance to all therapeutics that have been recommended for empirical treatment has emerged (1, 2), and only two drugs, namely, zoliflodacin (currently in phase 3 trials [3, 4]) and gepotidacin (in phase 2 trials [5, 6]), are in development as alternative options. Bioprospecting for antibiotics produced by microbes in soil communities could uncover novel inhibitory compounds against the gonococcus and other important human pathogens (7, 8). This approach can be implemented in undergraduate classrooms as an inquiry-based exercise, which was previously demonstrated by the Small World Initiative (9, 10), Tiny Earth (11, 12), and academic groups (13–16). Developed protocols screen for soil bacteria that produce antibiotics effective against “safe” bacteria (biosafety level 1 [BSL1]), which may also have inhibitory properties against pathogens within the same genus (e.g., ESKAPE pathogens [17, 18]). Here, we expand this methodology to *Neisseria*, using BSL1 commensals as proxies for pathogens, and identify three soil microbes (WAM01, WAM04, and WAM06) that inhibit commensal *Neisseria* growth as part of an undergraduate-level classroom exercise in the Thomas H. Gosnell School of Life Sciences at the Rochester Institute of Technology (RIT) (BIOL126-Introductory Biology Laboratory).

**FIG 1:**
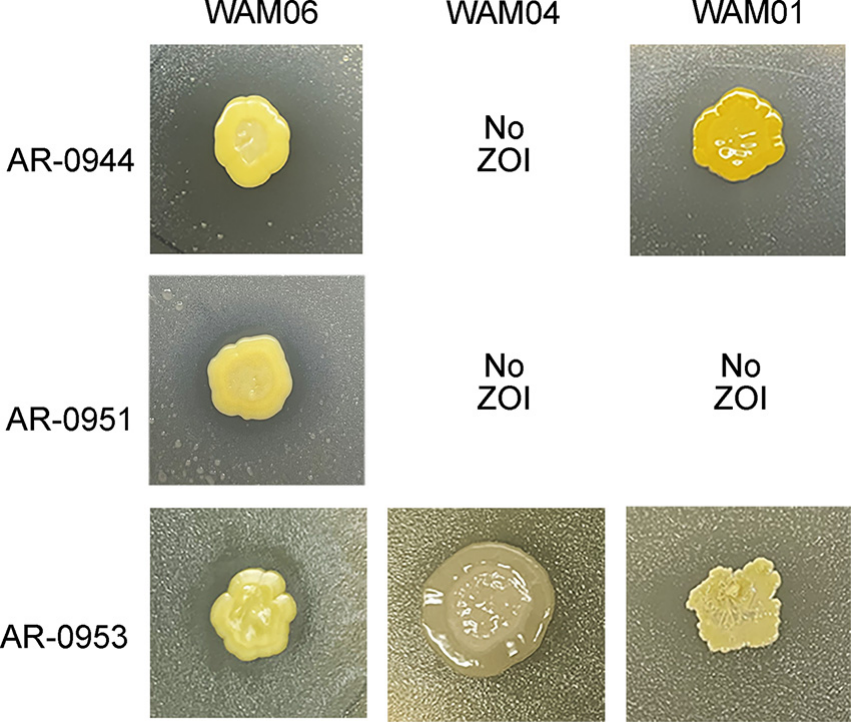
Competitive growth assay on 50% TSA for soil bacteria and commensal *Neisseria* strains. Micrococcus yunnanensis (WAM06) shows clear ZOIs against all three of the commensal *Neisseria* species tested. The Peribacillus butanolivorans (WAM04) isolate slightly inhibited the growth of one species, and the Staphylococcus pasteuri isolate inhibited the growth of two.

Soil samples were collected from Geneseo, New York (USA), and included sediment from an agricultural drainage ditch located at Big Tree Farm (42.798, −77.846) and soil from under an oak tree at the end of Main Street (42.792, −77.815). From these samples, serial dilutions were prepared on 50% tryptic soy agar (TSA), and individual colonies were isolated after 1 week of incubation at room temperature. Commensal *Neisseria* strains were obtained from the Centers for Disease Control and Prevention (CDC) and Food and Drug Administration (FDA) AR Isolate Bank *Neisseria* species matrix-assisted laser desorption ionization–time of flight (MALDI-TOF) verification panel, including AR-0944 (Neisseria cinerea), AR-0951 (Neisseria mucosa), and AR-0953 (Neisseria subflava), which were previously characterized and their draft assemblies published (19). Commensal *Neisseria* strains were plated as a lawn on 50% TSA and were subsequently inoculated with a patch (1 cm by 1 cm) of the soil bacterial strains WAM01, WAM04, and WAM06. Resultant cocultures were incubated at 28°C for 1 week, and the presence or absence of zones of inhibition (ZOIs) was recorded (Table 1 and Fig. 1). WAM06 produced a ZOI against all commensal *Neisseria* strains tested.

**TABLE 1:** Strain attributes and genome assembly overview of the soil bacteria isolated in this study^*a*^

Strain	Isolation site	Species	ZOI with:			No. of reads	Total assembly length (bp)	No. of contigs	Coverage (×)	*N*_50_ (bp)	No. of coding domains	No. of tRNAs	GC content (%)	SRA accession no.	GenBank accession no.
			AR-0944 (Neisseria cinerea)	AR-0951 (Neisseria mucosa)	AR-0953 (Neisseria subflava)										
WAM01	Big Tree Farm ditch, Geneseo, NY	Staphylococcus pasteuri	+	−	+	3,795,320	2,563,252	34	444.20	520,728	2,541	63	31.49	SRR19571305	JAMWYJ000000000
WAM04	Main Street oak, Geneseo, NY	Peribacillus butanolivorans	−	−	+	3,332,040	5,994,962	213	166.74	84,680	5,885	82	38.03	SRR19571306	JAMWYI000000000
WAM06	Main Street oak, Geneseo, NY	Micrococcus yunnanensis	+	+	+	2,289,342	2,127,231	249	322.86	23,464	2,038	41	72.5	SRR19571307	JAMWYH000000000

a All assembly statistics are based on contigs of ≥500 bp.

After incubation for 1 week at room temperature on 50% TSA, DNA was purified from isolates using the Thermo Fisher Scientific PureLink genomic DNA minikit after lysis in Tris-EDTA buffer with 0.5 mg/mL lysozyme and 3 mg/mL proteinase K. The Illumina Nextera XT kit was used to prepare libraries, which were pooled and sequenced using a 600-cycle v3 cartridge (2 × 300 bp) on the Illumina MiSeq platform at the RIT Genomics Core. Default parameters were used for all analyses except where otherwise noted. Paired-end sequencing resulted in an average of 3.13 ± 0.77 million reads, with an average read length of 185.63 ± 50.25 per library. Library quality was assessed using FastQC v0.11.9 (20), and SPAdes v3.14.1 (21) was used for *de novo* assembly. Assembly statistics were generated with QUAST (http://quast.sourceforge.net/quast), excluding contigs of <500 bp, and are reported in Table 1. Open reading frames (ORFs) were annotated using the GenBank Prokaryotic Genome Annotation Pipeline (PGAP) v5.2 (22) (Table 1), which was also used to assign genera and species, as follows: WAM01, Staphylococcus pasteuri; WAM04, Peribacillus butanolivorans; WAM06, Micrococcus yunnanensis. Further characterization of the anti-*Neisseria* compounds produced by the bacteria reported here will be reported in a future publication.

### Data availability.

The genome assemblies and raw reads are available in GenBank and the SRA, respectively, under the accession numbers listed in Table 1. All code is accessible at https://github.com/wadsworthlab/2022-soil-bacteria.
